# Supplementary material for: Unraveling the Transcriptional Dynamics of NASH Pathogenesis Affecting Atherosclerosis
Source: Int J Mol Sci. 2022 Jul 26;23(15):8229. doi: 10.3390/ijms23158229 (PMC9331250; doi:10.3390/ijms23158229)
Supplement: Supplementary file 1 [file ijms-23-08229-s001.zip › ijms-1745619-supplementary.pdf]

International Journal of
Molecular Sciences

MDPI

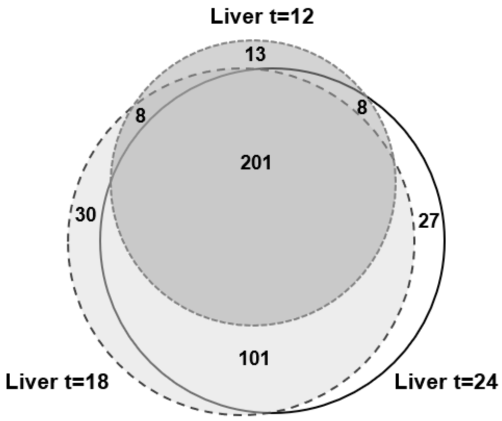

Figure S1. Venn diagram showing the overlap of DEPs in liver in Ldlr−/−.Leiden mice after 12, 18 and 24 weeks on HFD (*n* = 15) vs. chow diet (*n* = 6).

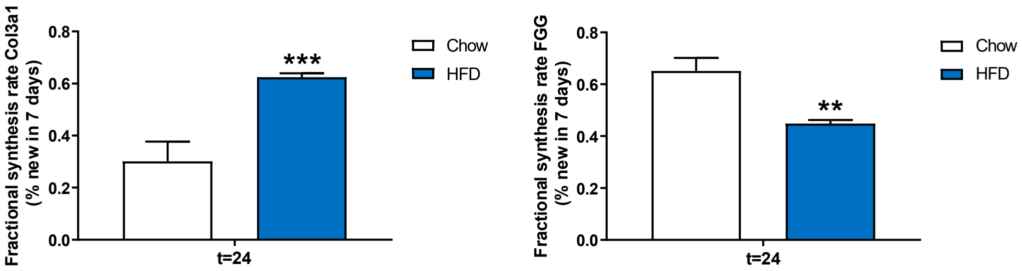

Figure S2. Gene expression of hepatic key regulators Col3a1 and FGG was confirmed on protein level in Ldlr−/−.Leiden mice after 24 weeks on HFD (*n* = 6) vs. chow diet (*n* = 3) by measuring the fractional synthesis rate using dynamic proteomics as previously described in more detail by van Koppen et al [29].
